# Response to letters by Baethge et al. and Martino et al.

**DOI:** 10.1186/s40345-017-0090-8

**Published:** 2017-05-31

**Authors:** Steven Marwaha, Andrew Thompson

**Affiliations:** 1 Unit of Mental Health and Wellbeing, Division of Health Sciences, Warwick Medical School, University of Warwick, Coventry, CV4 7AL UK; 2 Affective Disorders Service (IPU 3-8), Coventry, UK; 3 North Warwickshire Early Intervention in Psychosis Service, Coventry and Warwickshire NHS Partnership Trust, Nuneaton, UK

Dear Editor,

We thank Baethge et al. (2017) and Martino et al. (2017) for their interest and comments on our comprehensive literature review (Joyce et al. 2016). We focussed on answering the question of whether treatment given earlier in illness course is more effective than later. As we suggest in our review, this is a central tenet of the scientific basis of espousing early intervention in mental disorders including in bipolar disorders (Marwaha et al. 2016). The challenge is to identify methodologically robust evidence. Whilst we are cautious about the quality of current evidence, our review suggests that available treatments are more effective earlier in illness course. Our conclusions are based on ten studies included in the review. Eight studies, made up of two systematic reviews, and six incorporating primary evidence supported this conclusion. In 2 out of 10 of the included studies, this effect was rendered non-significant after adjusting for confounding.

Baethge et al. state that our results do not align with their own work (Baethge et al. 2003a, b;Bratti et al. 2003). They suggest that this discrepancy may be due to omissions in search terms that we used and secondly that our strategy of only including studies in which there was a direct comparison between people earlier and later in illness course is flawed because the latter group will include people who have poor prognosis or treatment resistance and the former with multiple prognoses. Martino et al. also focus on this latter point. Both letters suggest that the staging model of bipolar disorder is a hypothesis and not proven.

We agree with Baethge et al. and Martino et al. that the evidence base to investigate our question is limited in several ways, and we were of course at pains to point this out in the extensive limitations section of the paper. Indeed in assessing quality, we highlighted the risk of multiple biases that exist in most of the studies examined (see figure 2 in paper).

In terms of Baethge et al.’s first point, the main reason for this discrepancy may be that our review and their work, in essence, answer distinct though not necessarily wholly mutually exclusive questions. Our review was not concerned with latency or delay before first treatment but with effectiveness of treatment after the first (or first few) episode(s), and assessing the literature in relation to episode number and treatment (not necessarily prophylaxis) and effectiveness. In our strategy, we specifically avoided the issue of conflating latency or delay with episode number, in part because delay to first treatment can be lengthy but is highly variable (Dagani et al. 2016), and may encompass a range of previous affective symptoms and episodes (Berk et al. 2007; Howes et al. 2011). In addition, latency/delay may be just as much a function of the response of services as illness course. We also noted that in many studies first admission was aligned to first episode something which is unlikely to be the case and confounds analyses in this area. Whilst of course there may be an association between latency/delay and episode number, the nature of this is unlikely to be linear. Indeed in their own work, Baethge et al. (2003a) found that these two parameters are distinct, in that they report *“A greater therapeutic effect was demonstrated with increased severity of the illness prior to prophylaxis”*, (where illness severity was defined as hospitalisation rate before prophylaxis), whilst latency did not show a relationship. We agree that had we included the terms “delay” we could have extracted more overlapping papers included in their review (Baethge et al. 2003b) but it is likely that our protocol (developed before the work began) would have excluded these papers as they would not have met our inclusion criteria.

We agree that the work within Bratti et al. (2003) does concern our central question more so, though again the focus of that review appears to be pre-treatment episode number and its link to response to lithium treatment specifically. That paper concludes that there was not a clear and consistent signal. Our review covered both pharmacological (not only Lithium) and psychological treatments and found overall evidence in favour of the hypothesis that early treatment was more effective. Some discrepancy may be explained by the specificity of previous work focussing solely on Lithium and also its use as an acute treatment or in prophylaxis. Another reason for discrepancy may be that the evidence base that we interpreted contained five papers which were published after their review of 2003.

A major point raised by Baethge et al. as well as Martino et al., which we would partly agree with, is that those with multiple episodes have a worse prognosis than those with a first episode who would be made up of a group with varying prognoses (though unknown at the time of sampling). However, given the highly recurrent nature of bipolar disorder (Angst et al. 2003), it is likely that the vast majority of the first episode groups would be likely to go on to develop further episodes; for example, 50% relapse in as little as a year (Tohen et al. 2003). This reduces the validity of claims that the conclusions of our review are entirely dependent on comparing good vs poor prognosis groups. Some people in the multiple episode groups are likely to have been treatment resistant although how many would fulfil strict criteria of having failed two treatment attempts (Gitlin 2006) in the current episode is unclear. We also agree that the heterogeneity in the literature makes comparisons difficult and indeed it is for this reason we did not attempt a meta-analysis. We agree with the suggestion of Martino et al. that a longitudinal study examining effectiveness of treatment between many consecutive episodes experienced by the same patient would enable further understanding of this issue, though wonder how practicable this study design is.

Both Baethge et al. and Martino et al. raise the question of whether neuroprogression occurs within bipolar disorder and whether clinical staging is useful. We suggest in our paper that our results could be explained by this type of model, but agree that the evidence is not clear. In principle, we would take the view suggested by Scott et al. (2013) that clinical staging of bipolar disorder is a model with testable assumptions that require further study.

Finally, our esteemed colleagues all suggest that despite their doubts about the evidence, clinicians should continue to aim for early diagnosis and early treatment on ethical and clinical grounds. Whilst of course we agree, we cannot get away from the fact that especially in publically funded mental healthcare systems, clinical need and ethics are not the only actors on the stage. Policy decisions related to mental health service development require evidence that an approach is more clinically and cost-effective than the current framework. It is only by demonstrating this, whilst explaining the flaws in the literature, that a rationale for early intervention in bipolar disorder may be advanced. We hope that our review forms part of this developing evidence base, though clearly more work is needed.

In conclusion, whilst we agree with many of the comments on the methodological quality of the literature, our review conclusions are based on the available contemporaneous evidence. We suspect our differing results, and perhaps differing perspectives are likely to be due to incorporating this newer evidence and interpreting findings alongside current hypotheses within the clinical staging model of mental disorders.
